# Disintegration of Carbon Dioxide Molecules in a Microwave Plasma Torch

**DOI:** 10.1038/srep18436

**Published:** 2015-12-17

**Authors:** Hyoung S. Kwak, Han S. Uhm, Yong C. Hong, Eun H. Choi

**Affiliations:** 1 Department of Electrical and Biological Physics, Kwangwoon University, 20 Gwangun-ro, Nowon-Gu, Seoul 01897, Republic of Korea; 2 Plasma Technology Research Center, National Fusion Research Institute, 37 Dongjangsan-ro, Gunsan-si, Jeollabuk-do 54004, Republic of Korea

## Abstract

A pure carbon dioxide torch is generated by making use of 2.45 GHz microwave. Carbon dioxide gas becomes the working gas and produces a stable carbon dioxide torch. The torch volume is almost linearly proportional to the microwave power. Temperature of the torch flame is measured by making use of optical spectroscopy and thermocouple. Two distinctive regions are exhibited, a bright, whitish region of high-temperature zone and a bluish, dimmer region of relatively low-temperature zone. Study of carbon dioxide disintegration and gas temperature effects on the molecular fraction characteristics in the carbon dioxide plasma of a microwave plasma torch under atmospheric pressure is carried out. An analytical investigation of carbon dioxide disintegration indicates that substantial fraction of carbon dioxide molecules disintegrate and form other compounds in the torch. For example, the normalized particle densities at center of plasma are given by *n*_*CO2*_/*n*_*N*_ = 6.12 × 10^−3^, *n*_*CO*_/*n*_*N*_ = 0.13, *n*_*C*_/*n*_*N*_ = 0.24, *n*_*O*_/*n*_*N*_ = 0.61, *n*_*C2*_/*n*_*N*_ = 8.32 × 10^−7^, *n*_*O2*_/*n*_*N*_ = 5.39 × 10^−5^, where *n*_*CO2*_, *n*_*CO*_, *n*_*C*_, *n*_*O*_, *n*_*C2*_, and *n*_*O2*_ are carbon dioxide, carbon monoxide, carbon and oxygen atom, carbon and oxygen molecule densities, respectively. *n*_*N*_ is the neutral particle density. Emission profiles of the oxygen and carbon atom radicals and the carbon monoxide molecules confirm the theoretical predictions of carbon dioxide disintegration in the torch.

One of the most difficult problems in mankind is the global warming phenomenon, caused by an increase in the carbon dioxide concentration in the atmosphere. The major source of the carbon dioxide (CO_2_) is the burning of hydrocarbon fuel. There is a project^1^,^2^,^3^,^4^,^5^ called CCS (carbon dioxide capture^6^,^7^,^8^ in a burning system and store^9^,^10^,^11^), but its cost is formidably high. Carbon dioxide may dissociate through a reaction^12^,^13^ with oxygen and nitrogen atoms in air, but the densities of these atoms are very low at room temperature. The ocean can take carbon dioxide, but apparently this uptake^14^ is likely insufficient. Carbon dioxide was dissociated by recently laser beams^15^ at room temperature, but laser energy needed for a substantial amount of CO_2_ dissociation is very high. Therefore, the most practical means of reducing carbon dioxide may be the thermal dissociation^16^,^17^,^18^,^19^,^20^,^21^,^22^ of carbon dioxide molecules. In this context, we propose a method of carbon dioxide dissociation associated with carbon dioxide capture and utilization (CCU)^23^,^24^.

In this article, we present a carbon dioxide torch which makes use of microwaves and investigate the dissociation properties of carbon dioxide molecules in a high-temperature torch. A carbon dioxide torch can contain highly active species, such as electrons, ions, and radicals, which serve to enhance the chemical reaction rate, eliminating the need for catalysts during the processing of materials. The dissociation of carbon dioxide molecules at a high temperature produces oxygen atoms abundantly, which are very reactive. A conventional torch operated by arc-discharge processes may not be appropriate due to electrode erosion caused by oxidation. Although an inductively coupled plasma (ICP) in the range of radio-frequency is recently used in thermal processing fields, it is not efficient. The typical energy efficiency of ICP into the plasma is less than 50% and drops markedly at high power (>100 kW)^25^. For these reasons, the best solution would be to generate a pure carbon dioxide torch operated by microwaves without electrodes. The present article presents an in-depth study of the pure carbon dioxide torch and discusses its characteristics, including its temperature profile and the CO_2_ disintegration properties in the torch. The carbon dioxide torch exhibits two distinctive regions: a bright, whitish region of a high-temperature zone and a bluish, dimmer region of a relatively low-temperature zone. The bright, whitish region is a typical torch based on plasma species and the bluish, dimmer region is carbon monoxide (CO) recombining with oxygen.

## Results

### Disintegration of carbon dioxide molecules at high temperatures

Carbon dioxide molecules pass through an extremely high temperature torch where a local thermodynamic equilibrium (LTE) is assumed for T > 2000K. They then may disintegrate into various chemical compounds. However, we assume the disintegration of carbon dioxide molecules into carbon monoxide and oxygen atoms, i.e., CO_2_ → CO + O for simplicity in the initial analytical attempt. The enthalpy and entropy changes due to this reaction are found from a table^26^ to be ∆*H* = 530 kJ mol^−1^ and ∆*S* = 147 J mol^−1^ degree^−1^, respectively. The Gibbs free energy of the spontaneous disintegration is given by *G* = ∆*H − T*∆*S*; therefore, the disintegration temperature of the carbon dioxide molecules into carbon monoxide and oxygen atoms is calculated to be about *T* = ∆*H*/∆*S* = 3600K.

In reality, carbon dioxide decomposition is far more complicated than the above analysis of the Gibbs free energy. The dominant species after the decomposition of carbon dioxide are *C, O*, *CO*, *C*_2_, *O*_2_ and *CO*_*2*_. Thus, there are 19 reactions expected in total. However, the model calculation in the gas kinetics is concentrated on the dominant reactions in a hot chamber with a temperature exceeding 2000K. The first consideration is the carbon dioxide decomposition represented by the chemical reactions of


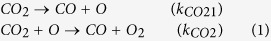


with their reaction constants^12^,^19^ of *k*_*CO21*_ = 2.14 × 10^−10^(*T*_*r*_*/T*)^0.5^exp(−52315/*T*) cm^3^ molecules^−1^ s^−1^, and *k*_*CO2*_ = 2.81 × 10^−11^ exp(−26458/*T*) cm^3^ molecules^−1^ s^−1^, where *T* is the gas temperature in Kelvin and *T*_*r*_ = 298 K represents the room temperature. The reaction constant^12^ *k*_*CO2*_ was obtained at the gas temperature *T* in the range of 300 K < *T* < 2500K. The constant *k*_*CO2*_ is estimated to be *k*_*CO2*_ = 7 × 10^−16^ cm^3^ molecules^−1^ s^−1^ at *T* = 2500K. On the other hand, the reaction constant^27^ of this reaction in equation (1) is *k*_*CO2*_ = 6.4 × 10^−15^ exp(−0084/*T*) cm^3^ molecules^−1^ s^−1^ in a different data set, being estimated as *k*_*CO2*_ = 2.06 × 10^−18^ cm^3^ molecules^−1^ s^−1^ at *T* = 2500K. The reaction constants in these two experimental data sets are very different from each other, even considering the different experimental conditions. Therefore, it may be difficult to use any of the data in this case. In this regard, we assume the reaction constant to be *k*_*CO2*_ = 3.56 × 10^−12^ exp(−26458/*T*) cm^3^ molecules^−1^ s^−1^, which ensures the disintegration of carbon dioxide molecules at *T* = 3600K, consistent with the simple analysis of the Gibbs free energy and thereby being a reasonable representation for the reaction constant in the subsequent model calculation of the gas kinetics. Nevertheless, the second reaction in equation (1) dominates the first reaction in the dissociation process of carbon dioxide, as will be shown later.

Carbon dioxide disintegration through the reactions in equation (1) may be balanced by carbon dioxide regeneration through the reaction


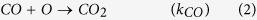


with their reaction constants^28^ of *k*_*CO*_ = 1.18 × 10^−13^(*T*_*r*_*/T*)exp(−3610/*T*) cm^3^ molecules^−1^ s^−1^. The reactions of equations (1) and (2) in the quasi-equilibrium state of the carbon dioxide density characterized by d*n*_*CO2*_/dt ≈ 0 lead to


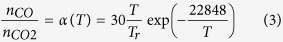


where *n*_*CO2*_ and *n*_*CO*_ are the densities of the carbon dioxide and the monoxide molecules, respectively. Here, quasi-equilibrium indicates that the reactions in equations (1) and (2) balance the number of carbon dioxide molecules at the local thermal equilibrium.

The second consideration is the generation of atomic carbon and its disappearance with the chemical reactions, expressed as


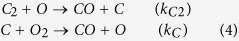


with the reaction constants^29^,^30^,^31^ of *k*_*C2*_ = 2.25 × 10^−11^ (*T/T*_*r*_) cm^3^ molecules^−1^ s^−1^ and *k*_*C*_ = 5.1 × 10^−11^(*T*_*r*_*/T*)^0.3^ cm^3^ molecules^−1^ s^−1^. Again, these reactions in the quasi-equilibrium state for the number of carbon atoms lead to


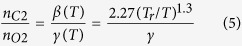


where *n*_*C2*_ and *n*_*O2*_ are the carbon and oxygen molecular densities and *γ* = *n*_*O*_/*n*_*C*_ is the ratio of the oxygen atom density *n*_*O*_ to the carbon atom density *n*_*C*_. The ratio *γ* will larger than unity throughout the range of gas temperatures. The number of carbon molecules in equation (5) is typically one order of magnitude lower than the number of oxygen molecules at a high gas temperature T > 2000K.

The third consideration is the generation of molecular oxygen and its disappearance with the chemical reactions, expressed as


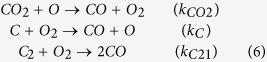


with the reaction constants^31^,^32^ of *k*_*CO2*_ = 3.56 × 10^−12^exp(−22848/*T*) cm^3^ molecules^−1^ s^−1^, *k*_*C*_ = 5.1 × 10^−11^(*T*_*r*_*/T*)^0.3^ cm^3^ molecules^−1^ s^−1^ and *k*_*C21*_ = 1.1 × 10^−11^exp(−381/T) cm^3^ molecules^−1^ s^−1^. The reactions in the quasi-equilibrium state for the molecular oxygen number lead to *k*_*CO2*_*n*_*CO2*_*n*_*O*_ = *k*_*C*_*n*_*C*_*n*_*O2*_ + *k*_*C21*_*n*_*C2*_*n*_*O2*_, which is further expressed as


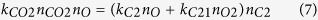


by identifying *k*_*C*_*n*_*C*_*n*_*O2*_ = *k*_*C2*_*n*_*C2*_*n*_*O*_ in equation (4). The next consideration is the generation of molecular carbon and its dissociation with the chemical reactions, expressed as


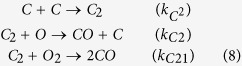


with the reaction constants^33^ of 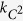 = 1.47 × 10^−11^(*T*_*r*_*/T*)^2.6^ cm^3^ molecules^−1^ s^−1^, *k*_*C2*_ = 2.25 × 10^−11^ (*T/T*_*r*_) cm^3^ molecules^−1^ s^−1^ and *k*_*C21*_ = 1.1 × 10^−11^exp(−381/T) cm^3^ molecules^−1^ s^−1^. The reactions in the quasi-equilibrium state for the molecular carbon number lead to


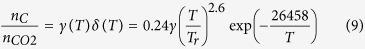


where the parameter *γ* = *n*_*O*_*/n*_*C*_ is the ratio of atomic oxygen and carbon densities.

Carbon dioxide molecules may disintegrate into five other species, including atomic oxygen and carbon. As will be shown later, the atomic numbers of carbon and oxygen at a gas temperature *T* higher than 2000K are more than three orders of magnitude higher than those of the molecules. Therefore, carbon dioxide molecules disintegrate mostly into oxygen *O* and carbon *C* atoms and carbon monoxide *CO* molecules. In this regard, the number *n*_*D*_ of carbon dioxide molecules which disintegrate may be related to the numbers, *n*_*O*_ and *n*_*CO*_, of oxygen and carbon monoxide molecules according to


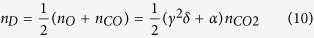


from equations (3) and (9). Similarly, the number *n*_*D*_ of disintegrated carbon dioxide molecules may also be related to the numbers, *n*_*C*_ and *n*_*CO*_, of carbon atoms and carbon monoxide molecules by


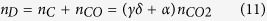


which also ensures the conservation of the number of carbon atoms. The solution to the relationship of *γ*^2^ + 2*γ* − *α/δ* = 0 from Eq. (10) and (11) is


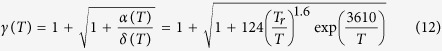


which determines the ratio of *γ* = *n*_*O*_*/n*_*C*_ in terms of the gas temperature *T*. We observe from Eq. (12) that the ratio *γ* = *n*_*O*_*/n*_*C*_ decreases from a large value to two as the gas temperature increases from room temperature to infinity, as expected. This indicates that all of the carbon dioxide molecules disintegrate into carbon and oxygen atoms at an extremely high temperature.

At this stage, we return to equation (7), checking carefully the relative strengths of the terms. The value *k*_*C2*_*n*_*O*_ + *k*_*C21*_*n*_*O2*_ on the right-hand side of equation (7) can be expressed as (*k*_*C2*_*γ*^2^*δ* + *k*_*C21*_*n*_*O2*_*/ n*_*CO2*_)*n*_*CO2*_. The reaction constant *k*_*C2*_ is typically one order of magnitude higher than *k*_*C21*_ at a practical gas temperature *T* in the range of 2000K < T < 7000K. We also note from equations (9) and (12) that the value of the parameter *γ*^2^*δ* is much larger than unity in this gas temperature range. The density ratio of *n*_*O2*_*/ n*_*CO2*_ is much less than unity, as will be shown later. The term proportional to *k*_*C21*_*n*_*O2*_ on the right-hand side of equation (7) can be neglected. In this context, equation (7) is simplified to


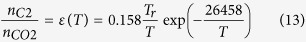


which is related to the density of *n*_*C2*_ to n_CO2_. Equations (3), (5), (9), (12), and (13) complete the relative density strengths of the gas species.

It is instructive to estimate the ratio of the molecular density to the atom density for carbon and oxygen. Making use of equations (5), (9) and (13), the density ratio is determined,


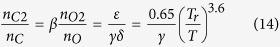


which indicates that the ratio of *n*_*C2*_*/n*_*C*_ is 10^−4^ or less at a gas temperature *T* higher than 2000K. Similarly, we also find from equations (5) and (14) that the density ratio of *n*_*O2*_*/n*_*O*_ is 10^−3^ or less at a high gas temperature. Thus, the derivation of the density ratio *γ* from equations (10) and (11) is well justified. We therefore conclude that most of the gas species in the microwave plasma torch with carbon dioxide gas are *CO*_2_ and *CO* molecules, with *C* and *O* atoms.

Carbon dioxide molecules disintegrate into atomic or bi-atomic molecules, increasing the number of particles. Designating *n*_*0*_ as the density of carbon dioxide before the disintegration step, it is shown as *n*_*0*_ = *n*_*CO2*_ + *n*_*D*_ = *n*_*CO2*_ + *n*_*CO*_ + *n*_*C*_ from equation (11). On the other hand, the total neutral density *n*_*N*_ may be approximated to be *n*_*N*_ = *n*_*0*_ + *n*_*O*_ = *n*_*CO2*_ + *n*_*CO*_ + *n*_*C*_ + *n*_*O*_ by neglecting the molecular densities. The carbon dioxide molecular density without any disintegration is the neutral density *n*_*0*_ = *n*_*N*_. Therefore, carbon dioxide dissociation increases the total number of particles and the gas volume. However, the neutral number density is determined by the gas temperature only for a one-atmospheric pressure torch. According to the ideal gas law, the total neutral density *n*_*N*_ can also be expressed as *n*_*N*_ = 2.6 × 10^19^ (*T*_*r*_/*T*) particles cm^−3^. This total neutral density *n*_*N*_ can equivalently be expressed as *n*_*N*_ = *n*_*CO2*_[1 + α + (1 + *γ*)*γδ*] by making use of equations (3) and (9). The densities of all gas species can then eventually be expressed as


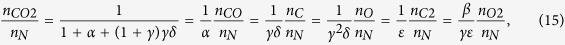


where the symbols *α, β, δ,* and *ε* are defined in equations (3), (5), (9) and (13), respectively. Equation (15) determines the densities of all of the species in the plasma torch in terms of the gas temperature *T*, thereby being one of the main results of this article.

**Figure 1:**
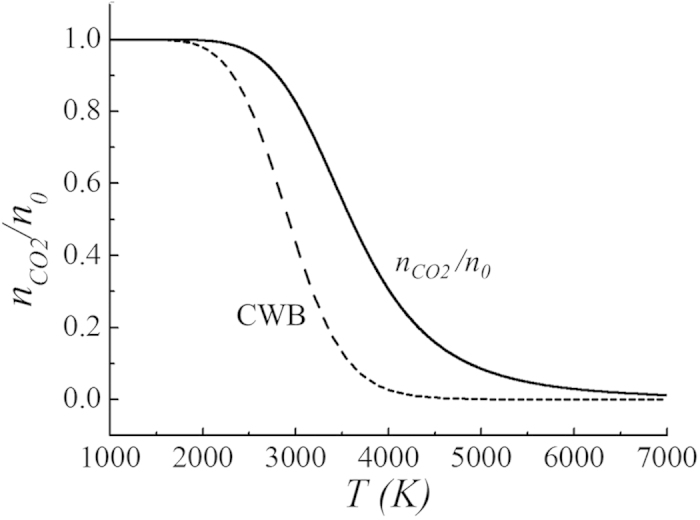
Plots of the density ratio *n*_*CO2*_/*n*_*0*_ versus the gas temperature *T* in Kelvin obtained from equation (15) (solid curve) from CWB code (dashed curve). The analytical results in Fig. 1 shows that the carbon dioxide molecules start to dissociate at about 2400K and that half of the molecules dissociate at *T* = 3600K, as expected from the disintegration analysis of the Gibbs free energy. Almost all the carbon dioxide molecules dissociate at *T* = 7000K except for a few percent. On the other hands, the numerical results from CWB code in Fig. 1 indicates that half of the molecules dissociate around *T* = 3100K, which is considerably lower than *T* = 3600K of the spontaneous dissociation temperature from the Gibbs free energy.

We neglected the first reaction in equation (1) during the derivation of equation (3), assuming that the second reaction in equation (1) dominates. The validity of this assumption is demonstrated by estimating the value of *k*_*CO21*_*n*_*N*_/*k*_*CO2*_*n*_*O*_, where *k*_*CO21*_ and *k*_*CO2*_ are defined in equation (1). After carrying out a straightforward calculation, it is shown as


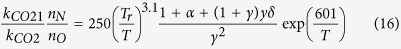


which is less than 10^−2^, ensuring the validity of the assumption made for the derivation of equation (3). In this regard, equation (15) will provide reasonably accurate information about the gas species in terms of the torch flame temperature *T* in the range of 2000K < *T* < 7000K. A simulation software tools named Chemical WorkBench (CWB), which was aimed at the reactor-scale kinetic modeling of homogeneous gas-phase, has been used for computation of species concentration. This software tool can be effectively used for the modeling, optimization, and design of a wide range of chemistry-loaded processes, including very high temperature. The CWB code is developed and distributed by Kintech Lab, Russia^34^. The analytical results from equation (15) can be compared with numerical results from CWB code for comparison. The number density of carbon dioxide molecules before decomposition is *n*_*0*_, as estimated by *n*_*0*_ = *n*_*CO2*_ + *n*_*CO*_ + *n*_*C*_. If carbon dioxide molecules begin to dissociate at a high temperature, the neutral density increases from *n*_*0*_ to *n*_*N*_. Shown in Fig. 1 are plot of the density ratio *n*_*CO2*_/*n*_*0*_ versus the gas temperature *T* in Kelvin obtained from equation (15) (solid curve) and CWB code (dashed curve). The analytical results in Fig. 1 shows that the carbon dioxide molecules start to dissociate at about 2400K and that half of the molecules dissociate at *T* = 3600K, as expected from the disintegration analysis of the Gibbs free energy. Almost all the carbon dioxide molecules dissociate at *T* = 7000K except for a few percent. On the other hands, the numerical results from CWB code in Fig. 1 indicates that half of the molecules dissociate around *T* = 3100K, which is considerably lower than *T* = 3600K of the spontaneous dissociation temperature from the Gibbs free energy. This discrepancy may be caused by differences between the chemical reaction constants used in the analytical calculation and numerical computations. Overall, the code results indicate easier dissociation of carbon dioxides than those of analytical results. Otherwise, trend of the analytical results in terms of the flame temperature agree reasonably well with those of numerical data.

**Figure 2:**
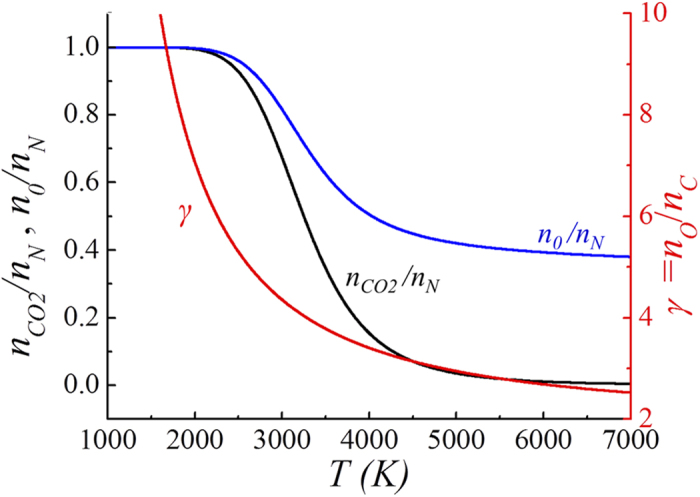
Plots of the ratio *γ* = *n*_*O*_*/n*_*C*_*, n*_*0*_*/n*_*N*_, and *n*_*CO2*_/*n*_*N*_ versus the gas temperature *T* in Kelvin. Plots of the ratio *γ* = *n*_*O*_*/n*_*C*_, the normalized initial (*n*_*0*_) and carbon dioxide molecular densities versus the gas temperature *T*. The ratio *γ* = *n*_*O*_*/n*_*C*_ is obtained from equation (12) and the normalized initial (*n*_*0*_*/n*_*N*_) and carbon dioxide (*n*_*CO2*_/*n*_*N*_) molecular densities are obtained from equation (15). It is also noted from Fig. 2 that the normalized initial density *n*_*0*_*/n*_*N*_ approaches 1/3 as the gas temperature becomes extremely high, indicating that the complete disintegration of CO_2_ into C and O atoms eventually increases the neutral number *n*_*N*_ until it is three times the original number *n*_*0*_.

Shown in Fig. 2 are plots of the ratio *γ* = *n*_*O*_*/n*_*C*_, the normalized initial (*n*_*0*_) and the carbon dioxide molecular densities versus the gas temperature *T*. The ratio *γ* = *n*_*O*_*/n*_*C*_ is obtained from equation (12) and the normalized initial (*n*_*0*_*/n*_*N*_) and carbon dioxide (*n*_*CO2*_/*n*_*N*_) molecular densities are obtained from equation (15). The ratio *γ* decreases to about 2 as the gas temperature *T* increases. It is also noted from Fig. 2 that the normalized initial density *n*_*0*_*/n*_*N*_ approaches 1/3 as the gas temperature becomes extremely high, indicating that the complete disintegration of CO_2_ into C and O atoms eventually increases the neutral number *n*_*N*_ until it is three times the original number *n*_*0*_.

**Figure 3:**
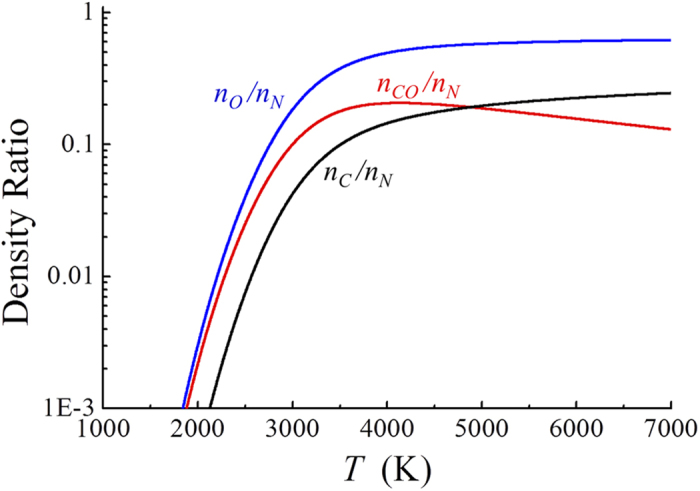
Plots of oxygen and carbon atom densities versus the gas temperature *T* obtained from equation (15). The oxygen and carbon atom densities increase drastically as the gas temperature exceeds *T* = 4200K by the dissociation of carbon monoxide. A normalized carbon monoxide molecule is also presented here for comparison with other species. Several points are noteworthy in Fig. 3. First, the oxygen and carbon atom densities are substantial fractions of the neutral density at a high temperature. Seconds, the oxygen atom density is less than three times the carbon atom density throughout the high-temperature range (*T* > 4500K) indicating the dissociation of a substantial fraction of carbon monoxide molecules. Third, the oxygen atom density is always higher than the carbon monoxide density throughout the range of gas temperatures.

**Figure 4:**
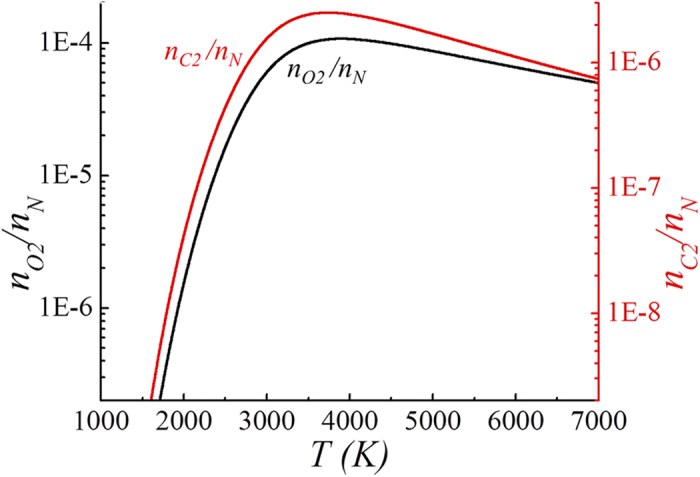
Plots of the normalized oxygen and carbon molecular densities versus the gas temperature *T* obtained from equation (15). The oxygen and carbon molecular densities are several orders of magnitude lower than the oxygen and carbon atom densities. It is also observed in Fig. 4 that the oxygen and carbon molecular densities decrease as the gas temperature *T* increases from *T* = 4000K, indicating the dissociation of molecules at a high temperature.

The oxygen and carbon atom densities increase drastically as the gas temperature exceeds *T* = 4200K by the dissociation of carbon monoxide. Figure 3 presents plots of the oxygen and carbon atom densities versus the gas temperature *T* obtained from equation (15). A normalized carbon monoxide molecule is also presented in Fig. 3 for comparison with other species. Several points are noteworthy in Fig. 3. First, the oxygen and carbon atom densities are substantial fractions of the neutral density at a high temperature. For example, the normalized particle densities at *T* = 4500K are given by *n*_*O*_/*n*_*N*_ = 0.55, *n*_*C*_/*n*_*N*_ = 0.175 and *n*_*CO*_*/n*_*N*_ = 0.2, which also satisfy the condition *n*_*O*_ + *n*_*CO*_ = 2(*n*_*C*_ + *n*_*CO*_), in order to conserve the oxygen and carbon numbers. Seconds, the oxygen atom density is less than three times the carbon atom density throughout the high-temperature range (*T* > 4500K) indicating the dissociation of a substantial fraction of carbon monoxide molecules. Third, the oxygen atom density is always higher than the carbon monoxide density throughout the range of gas temperatures. The dissociation of carbon monoxide is outstanding at gas temperatures higher than T = 4800K, beyond which even the carbon atom density dominates over the carbon monoxide density. Finally, we also note from the numerical data obtained from equation (15) that the oxygen atom and carbon monoxide densities remain high even at relatively low temperatures. For example, the oxygen atom and carbon monoxide densities at *T* = 1500K are given by *n*_*O*_ = 2.41×10^14^ molecules cm^−3^ and *n*_*CO*_ = 1.98 × 10^14^ molecules cm^−3^, ensuring active chemical reactions in this temperature range. Shown in Fig. 4 are plots of the normalized oxygen and carbon molecular densities versus the gas temperature *T* obtained from equation (15). As expected from equation (14), the oxygen and carbon molecular densities are several orders of magnitude lower than the oxygen and carbon atom densities. It is also observed in Fig. 4 that the oxygen and carbon molecular densities decrease as the gas temperature *T* increases from *T* = 4000K, indicating the dissociation of molecules at a high temperature.

The density *n*_*CO*_ of the carbon monoxide molecules for a specified value of *n*_*CO*_*/n*_*N*_ in equation (15), for example, is proportional to the neutral density *n*_*N*_, which is inversely proportional to the gas temperature *T* according to the equation of state for an ideal gas. Here we assumed that all chemical species are in the condition of blackbody radiation. Therefore, the carbon monoxide density is expressed as *n*_*CO*_ = *n*_*N0*_(*x/x*_*0*_)(*T*_*0*_*/T*), where *x*_*0*_ and *n*_*N0*_ are the normalized *CO* and neutral densities, respectively, at an arbitrary temperature *T*_*0*_. Here, *x* = *n*_*CO*_/*n*_*N*_. Assuming a local thermal equilibrium, the net light-intensity emitted at the characteristic wavelength of carbon monoxide molecules is proportional to the mean energy density *u*_*CO*_ of a photon^35^, which is proportional to *T*^4^. The mean energy density *u*_*CO*_ of a photon at this characteristic wavelength may also be proportional to the carbon monoxide density *n*_*CO*_. Thus, the normalized light intensity *I*_*CO*_ can be expressed as *I*_*CO*_ = (*x/x*_*0*_)(*T/T*_*0*_)^3^, which is normalized by the light intensity at the temperature *T*_*0*_. Making use of the normalized densities, we can estimate the normalized light emission intensities of all of the gas species obtained from equation (15).

**Figure 5:**
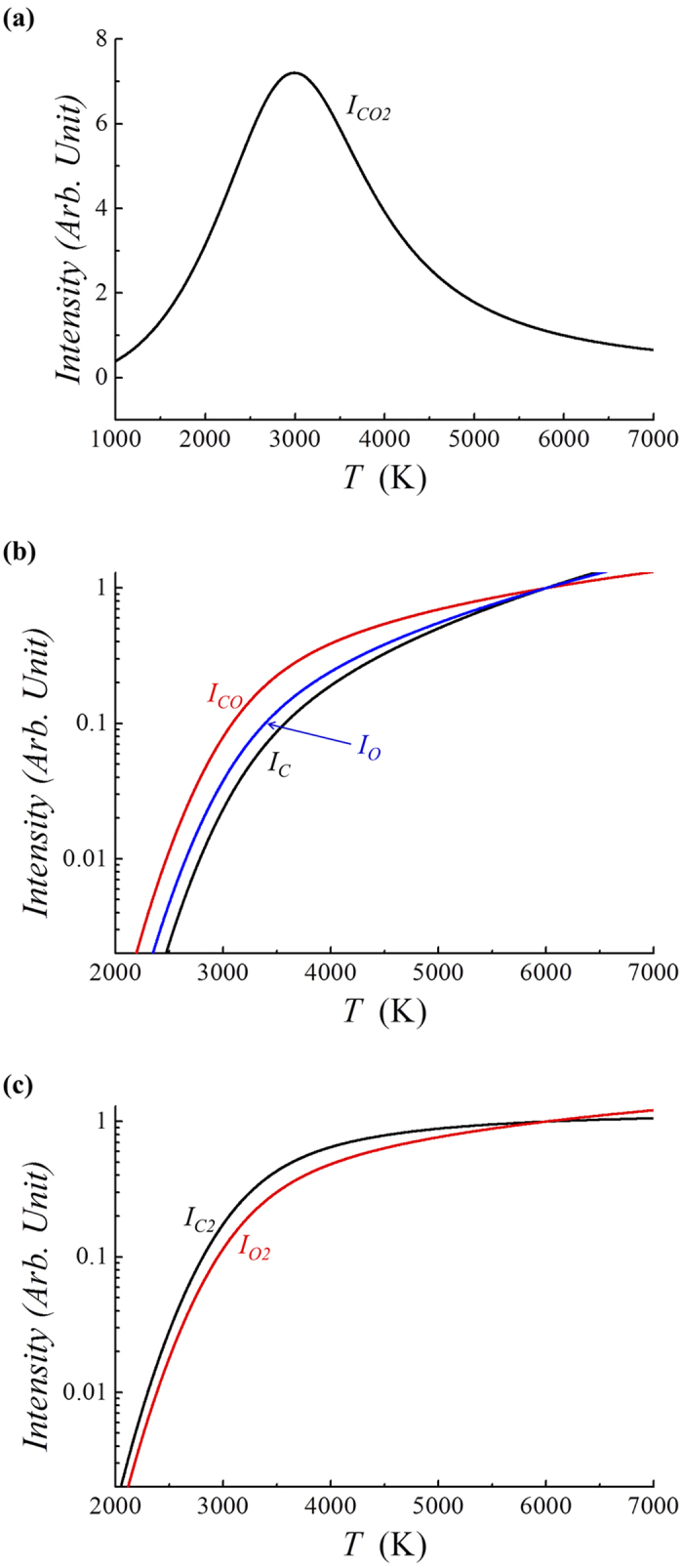
Plots of the light emission intensities of (a) *I*_*CO2*_, (b) *I*_*CO*_, *I*_*O*_ and *I*_*C*_, and (c) *I*_*C2*_ and *I*_*O2*_ versus the gas temperature *T* in Kelvin. The light intensities are normalized by those at the temperature *T*_*0*_ of 6000K. Several points are noteworthy from Fig. 5. First, the intensity of the carbon dioxide emission increases drastically, reaches its peak at around *T* = 3000K, and then decreases as the gas temperature *T* increases from 1000K. The decrease in the CO_2_ light emission at a high temperature is caused by the decrease in the molecular density of carbon dioxide due to dissociation at a high temperature. Second, the light emission of all other species increases as the gas temperature *T* increases because their densities increase. Third, the emission intensities of oxygen and carbon molecules increase relatively slowly at a high gas temperature beyond *T* = 3500 K as the gas temperature increases.

The light emission intensity can be calculated relatively in terms of the light emission at *T* = *T*_*0*_. The normalized light intensities *I*_*CO*_ at the characteristic wavelength of carbon monoxide, for example, can be calculated in terms of the gas temperature in Fig. 5b. Plotted in Fig. 5 are the light emission intensities calculated theoretically versus the gas temperature *T* in Kelvin. The light intensities are normalized by those when the temperature *T*_*0*_ equals 6000K. Several points are noteworthy from Fig. 5. First, the intensity of the carbon dioxide emission increases drastically, reaches its peak at around *T* = 3000K, and then decreases as the gas temperature *T* increases from 1000K. The decrease in the CO_2_ light emission at a high temperature is caused by the decrease in the molecular density of carbon dioxide due to dissociation at a high temperature. Second, the light emission of all other species increases as the gas temperature *T* increases because their densities increase. Third, the emission intensities of oxygen and carbon molecules increase relatively slowly at a high gas temperature beyond *T* = 3500K as the gas temperature increases.

## Experimental Results

**Figure 6:**
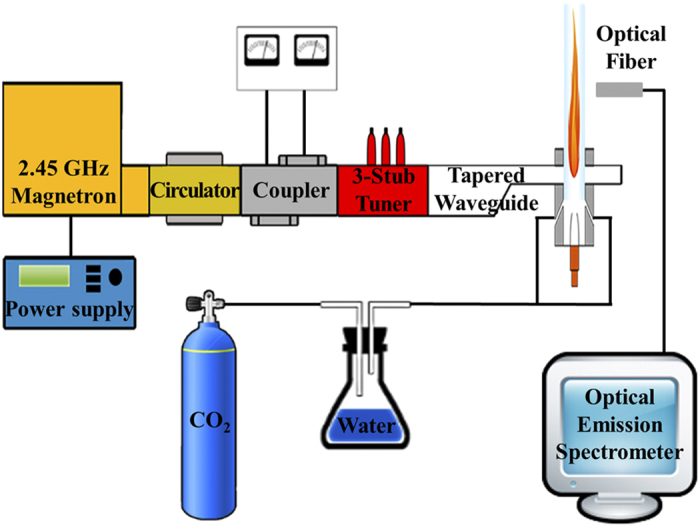
Schematic presentation of the microwave plasma torch system for the generation of the carbon dioxide torch and optical emission spectroscopy system. The torch system consist of a 2.45 GHz magnetron, a power supply, a WR-340 waveguide components, including a circulator, a coupler, a 3-stub tuner and a tapered waveguide. In this study, we mixed 100 sccm of water vapor into 10 lpm of carbon dioxide gas for gas temperature determination from OH emissions. Also, this figure shows a schematic of the experimental setup for optical emission spectroscopy system. The emission light of carbon dioxide torch is captured by optical fiber and analyzed by a spectrometer and monochromator. We used HR-4000(Ocean optics) spectrometer and VM-505(Acton) monochromator.

**Figure 7:**
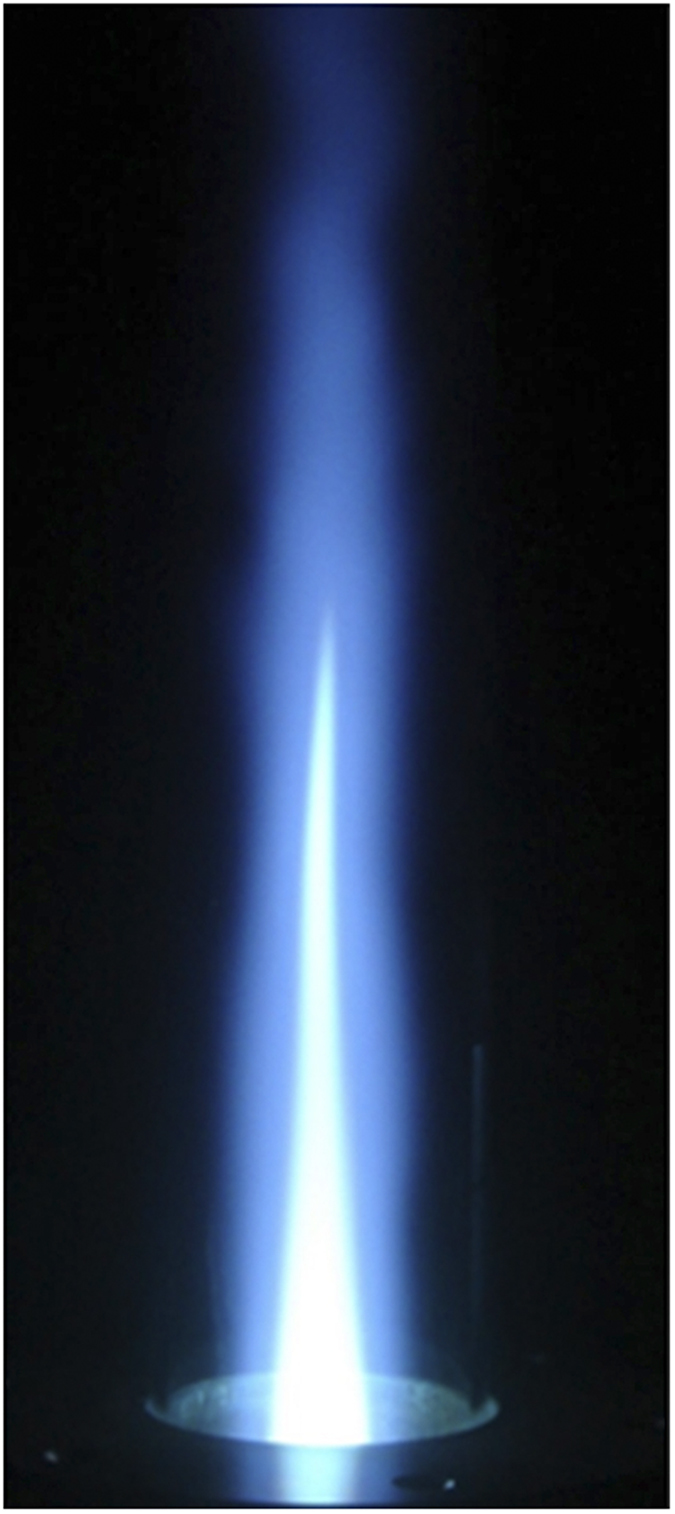
The flame of carbon dioxide plasma torch. Typical carbon dioxide torch shows inside a quartz tube with a 3 cm diameter and a length of 30 cm. The torch is powered by 2 kW of microwave power. The torch volume is almost linearly proportional to the microwave power. The carbon dioxide torch exhibits two distinctive regions: a bright, whitish region of a high-temperature zone and a bluish, dimmer region of a relatively low-temperature zone. The bright, whitish region is a typical torch based on plasma species and the bluish, dimmer region is carbon monoxide recombining with oxygen.

**Figure 8:**
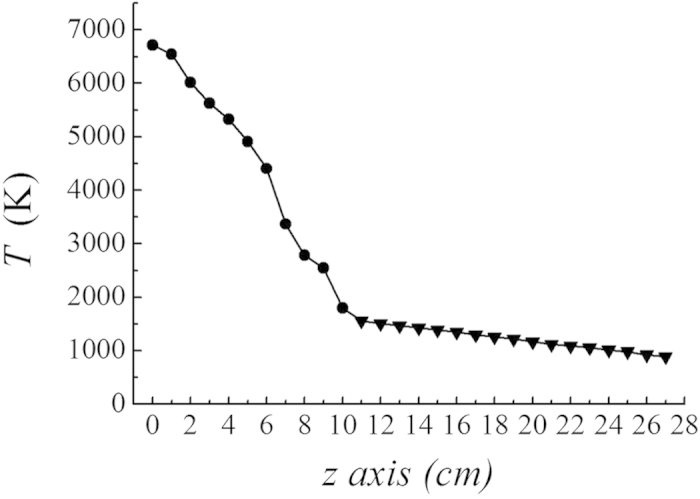
The temperature distribution of carbon dioxide plasma torch. The measured data of the flame temperature *T* versus the axial distance *z* from the center of the plasma flame at 2 kW. The temperature of the torch flame was measured by making use of a thermocouple device (*T* < 2000 K) and optical emission spectroscopy (*T* > 2000 K).

Figure 6 presents the microwave plasma torch system for the generation of the carbon dioxide torch. The design and operation of the atmospheric microwave-plasma torch are briefly summarize at Methods section for completeness, although they have been reported in detail in previous literature^36^. A typical example of the carbon dioxide plasma torch is presented in Fig. 7, in which the carbon dioxide torch is shown inside a quartz tube which has a 3 cm diameter and a length of 30 cm. The plasma torch shown in Fig. 7 is operated by 2 kW of microwave power. Measuring the gas temperature along the axis of the plasma flame is important for the characterization of the carbon dioxide torch at atmospheric pressure (See Method section). The emission spectrum of carbon dioxide torch mainly consist of *C*_*2*_ swan band^37^ and other emitter system including *C* and *O* atom, *OH*, *CN*, *CO*, etc. Shown in Fig. 8 is the measured data of the flame temperature *T* versus the axial distance *z* from the center for the 2 kW plasma flame. It is clear from Fig. 8 that there are two regions of the carbon dioxide flame, a high-temperature zone and a relatively low temperature zone. The torch flame of the high-temperature zone ranging from *z* = 0 to *z* = 10 cm is white and bright due to light emitted from various species at high-temperature, as it is a typical emission of a high-temperature plasma. Meanwhile, the flame color of parts of the low-temperature zone beyond *z* = 10 cm becomes bluish due to light emitted from excited carbon monoxide Herzberg system(C^1^∑→A^1^Π) and fourth positive system(A^1^Π→X^1^∑)^38^, characteristic of CO recombining with oxygen. It is useful in the subsequent analysis to estimate the flow rate of carbon dioxide in the quartz tube with its inner diameter of 2.6 cm. Assuming 10 liter per minute (lpm) of carbon dioxide gas and the ideal gas law, the flow rate at *T* = 6000 K was calculated to be 620 cm s^−1^.

**Figure 9:**
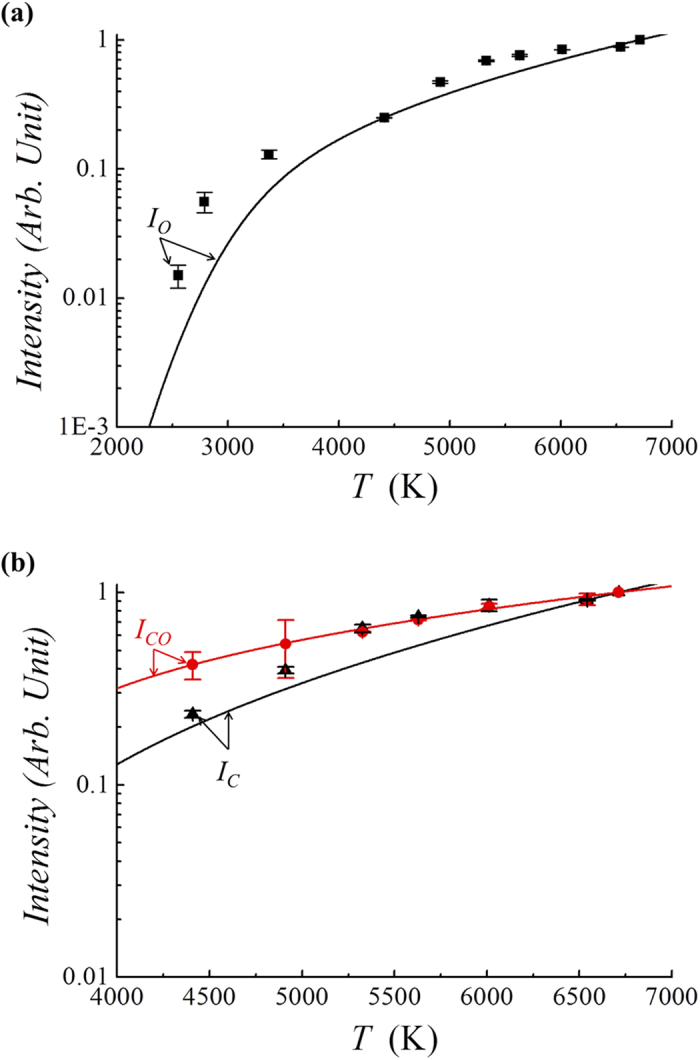
The theoretical normalized light intensities in comparison with experimental data. This figure shows the plots of the normalized light intensities (**a**) *I*_*CO*_ (circular dots) at 269 nm for carbon monoxide and *I*_*C*_ (triangular dots) at 909 nm for atomic carbon, as well as (**b**) *I*_*O*_ (square dots) at 777 nm for atomic oxygen versus the temperature *T* in Kelvin. The curves are the theoretically predicted values, which are similar to Fig. 9(b) except for the temperature *T*_*0*_ = 6710K, corresponding to normalization.

The light emission intensity can be measured relatively in terms of the light emission at *T* = *T*_*0*_. The normalized light intensities *I*_*CO*_ (circular dots) at 269 nm for carbon monoxide and *I*_*C*_ (triangular dots) at 909 nm for atomic carbon in Fig. 9(a), and *I*_*O*_ (square dots) at 777 nm for atomic oxygen in Fig. 9(b), are presented in Fig. 9 in terms of the temperature *T* in Kelvin. The light intensities are normalized by those at the temperature *T*_*0*_ of 6710K, the gas temperature at the center of the torch (z = 0). The curves are the theoretically predicted values, a finding similar to that in Fig. 5(b) except for the temperature *T*_*0*_ = 6710K corresponding to normalization. The upper curve in Fig. 9(a) corresponds to CO. The emission intensities of CO, O and C decrease drastically as the gas temperature *T* decreases from *T* = 6710 K. The emission profiles of the experimental data at a high temperature are in reasonably good agreement with the theoretical predictions. However, the emission intensity measurements of CO and C radical light in a relatively low-temperature range (*T* < 4000 K) are very difficult due to the noise caused by flame fluctuations.

## Discussion

A pure carbon-dioxide torch was generated by making use of 2.45 GHz of microwave energy. The generation of the carbon dioxide plasma torch was described in Method section, which describes how the carbon dioxide gas enters the discharge tube as a swirl gas at room temperature. The carbon dioxide plasma torch was very stable and can typically operate until the microwave power is deactivated. The temperature of the torch flame was measured by making use of a thermocouple device and optical spectroscopy. It exhibits two distinctive regions: a bright, whitish region of a high-temperature zone and a bluish, dimmer region of a relatively low-temperature zone. The bright, whitish region is a typical torch based on plasma species and the bluish, dimmer region is carbon monoxide recombining with oxygen. The properties of carbon dioxide disintegration were analytically investigated in Result section, showing that a substantial fraction of carbon dioxide disintegrates and forms other compounds at high temperatures in the carbon dioxide torch. For example, the normalized particle densities at center of plasma (*T* = 6710K) are given by *n*_*CO2*_/*n*_*N*_ = 6.12 × 10^−3^, *n*_*CO*_/*n*_*N*_ = 0.13, *n*_*C*_/*n*_*N*_ = 0.24, *n*_*O*_/*n*_*N*_ = 0.61, *n*_*C2*_/*n*_*N*_ = 8.32 × 10^−7^, *n*_*O2*_/*n*_*N*_ = 5.39 × 10^−5^, respectively. The emission profiles of the carbon monoxide molecules, carbon atoms and oxygen atoms confirm the theoretical predictions of the carbon dioxide disintegration in the carbon dioxide torch.

A high-temperature carbon-dioxide torch has the potential to be applied to hydrocarbon fuel reforming at one atmospheric pressure. As an example, we consider the reforming of methane according to CO_2_ + CH_4_ *→* 2CO + 2H_2_. The enthalpy and entropy changes due to this reaction can be calculated from data in a readily available table^24^ to be Δ*H* = 247 kJ mol^−1^ and Δ*S* = 257 J mol^−1^, respectively. The Gibbs free energy of the reaction is given by *G* = Δ*H−T*Δ*S*; therefore, the reaction temperature for reforming is calculated to be *T* = Δ*H*/Δ*S* = 961K. The temperature of the carbon dioxide plasma torch shown in Fig. 8 is much higher than the reaction temperature *T* = 961K in most torch flames at atmospheric pressure. Moreover, radicals including carbon and oxygen atoms are abundantly available in the carbon dioxide torch, dramatically enhancing the reaction speed. Methane may also break down at high temperatures. On the other hand, methane reforming in a conventional steam system may need various catalytic materials, which are very frequently contaminated by carbon deposition, deteriorating the conversion efficiency. There are many other potential applications of the carbon dioxide torch developed here.

## Method

### Generation of the carbon-dioxide plasma torch

The magnetron in Fig. 6 receives electrical power from a power supply, generating 2.45 GHz microwaves, which propagate through a circulator, a power meter, a three-stub turner and a tapered waveguide, entering a discharge tube made of quartz. The gas feeder provides a swirl gas of carbon dioxide in the discharge tube, creating a vortex flow, stabilizing the torch flame in the center of the tube and protecting the discharge tube from the heat of the torch. Microwaves from the magnetron propagate through the waveguide, concentrating their power in the tube and generating a plasma torch with a temperature of 6000 K and a plasma density on the order of 10^13^ particles cm^−3^. Once the plasma torch is ignited, nearly 100% of the microwave power is absorbed by the torch plasma with a reflected wave power of less than 1%.

The quartz discharge tube sits on the gas feeder through a steel tapered waveguide. Carbon dioxide gas enters the discharge tube though the gas feeder before the ignition of the plasma. The CO_2_ plasma torch is very stable and can usually operate until the carbon dioxide in a gas tank is consumed.

### Measurement of the carbon-dioxide plasma torch temperature

The carbon-dioxide flow rate in Fig. 7 was 10 lpm, and approximately 0.1 lpm of water gas entered the discharge tube for OH emission at 309 nm, which provides vital information about the gas temperature in the torch. After a careful investigation of the flame diameter and the flame length, we found that the flame volume increases almost linearly with the electrical power.

**Figure 10:**
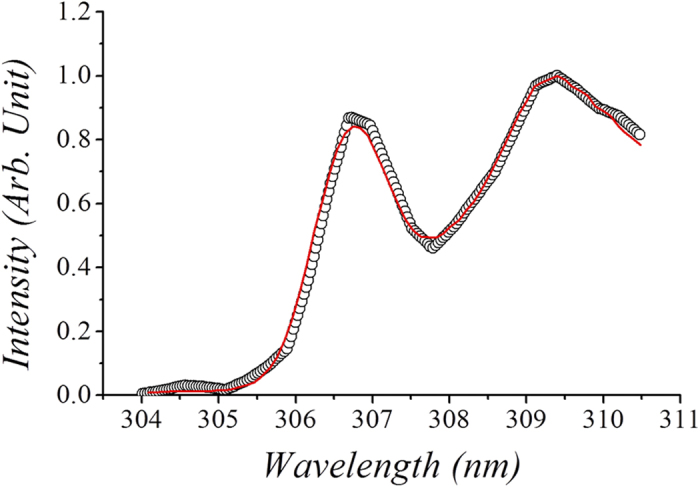
Profile of the simulated optical emission of OH radicals. The optical emission data was analyzed through a computer code named SPECAIR, which is a program for modeling the absolute intensity spectral radiation emitted by gases and plasmas of various compositions such as molecular transitions of C_2_, CO, CN, NO, N_2_, N_2_^+^, OH, NH, O_2_ and atomic lines of C, N, O. Profile of the simulated optical emission of OH radicals around 309 nm as represented by the solid curve in comparison with the experimental emission data (open circular dots), estimating the gas temperature to be *T* = 4912 K.

A high gas temperature was estimated by making use of an optical spectrum, as shown in Fig. 10, where the experimental data of the optical signals were obtained through an optical fiber placed near a specified portion of z in the plasma torch. Here, z represents the distance from the center of the torch, where the base of the torch is located inside the waveguide, as shown in Fig. 6, designating z = 0 as the center of the flame, which is the midpoint of the waveguide. The experimental data in Fig. 10 are the optical emission of the hydroxyl molecules from the position of z = 5 cm in the plasma torch. The optical emission data of each portion of the torch flame were digitalized and stored in a computer. In order to obtain the spectrum of the hydroxyl molecules, we mixed 100 sccm of water vapor into 10 lpm of carbon dioxide gas. The experimental data in Fig. 10 at z = 5 cm are an example of the optical emission of OH radicals, which is related to the rotational structure of diatomic gases, providing information regarding the rotational temperature. Molecules are in the rotational states and neutral gas molecules are at thermal equilibrium due to the low energies needed for rotational excitation and the short transition times. Therefore, the gas temperature can be obtained from the rotational temperature^39^.

The optical emission data was analyzed through SPECAIR computer code, which is a program for computing, manipulating and fitting spectra. This program can automatically identify the species in spectrum and enables modeling of the absolute intensity of radiation emission from gases and plasmas of various compositions in the wide range of wavelength for different density. SPECAIR uses database of an initial LTE species distribution in air at various temperatures which include electronic, vibrational, rotational and translational excitations in the wide temperature range^40^.

The profile of the simulated optical emissions^41^ of OH radicals around 309 nm according to the SPECAIR computer code, represented by the solid curve in Fig. 10, is compared with the experimental emission data, estimating the gas temperature to be *T* = 4900 K. As an example, the gas temperature along the plasma flame generated by 2 kW of microwave power was measured. The torch flame used to measure the temperature is very similar to the flame shown in Fig. 7. A flame temperature up to *T* = 2000 K can be measured by making use of a thermocouple device. On the other hand, the flame temperature higher than *T* = 2000 K is measured by means of optical spectroscopy, as noted above.

## Additional Information

**How to cite this article**: Kwak, H. S. *et al.* Disintegration of Carbon Dioxide Molecules in a Microwave Plasma Torch. *Sci. Rep.* **5**, 18436; doi: 10.1038/srep18436 (2015).
